# Supplementary material for: Organization of corticocortical and thalamocortical top-down inputs in the primary visual cortex
Source: Nat Commun. 2024 May 27;15:4495. doi: 10.1038/s41467-024-48924-8 (PMC11130321; doi:10.1038/s41467-024-48924-8)
Supplement: Supplementary file 1 — Supplementary Information [file 41467_2024_48924_MOESM1_ESM.pdf]

Supplementary Information

Organization of Corticocortical and Thalamocortical Top-down Inputs in the Primary Visual Cortex

Yanmei Liu[1,2§], Jiahe Zhang[1,2§], Zhishan Jiang[1,2], Meiling Qin[3], Min Xu[3], Siyu Zhang[1,2]*, Guofen Ma[1,2]*

*Corresponding author. E-mail: zhang_siyu@sjtu.edu.cn (S. Z.), guofenma@sjtu.edu.cn (G. M.)

Supplementary figures and legends

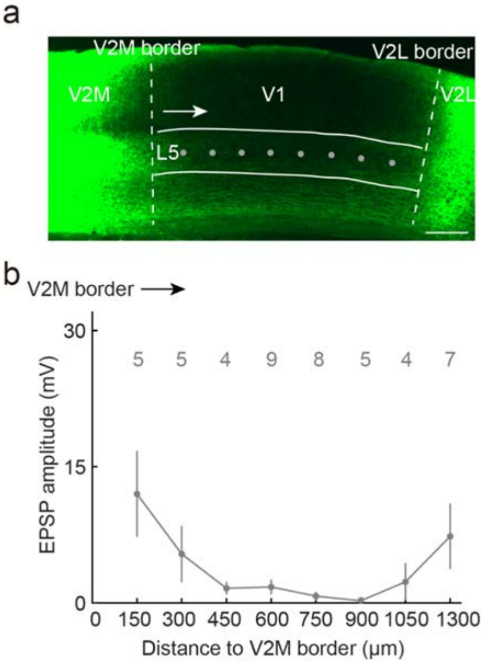

Supplementary Fig. 1. Strengths of V2M inputs in relation to the distance between the recorded V1 L5 Pyrs and the V2M border. a, Fluorescent image showing the distribution of V2M axons (green) in V1. Gray dots represent the recording sites of V1 Pyrs. Scale bar, 200 μm. **b**, EPSC amplitude in V1 L5 Pyrs evoked by activation of V2M inputs. The number of neurons in each position is indicated by the numbers displayed in the figure. n = 3 mice, 3slices. Data are presented as the mean ± SEM. Source data are provided as a Source Data file.

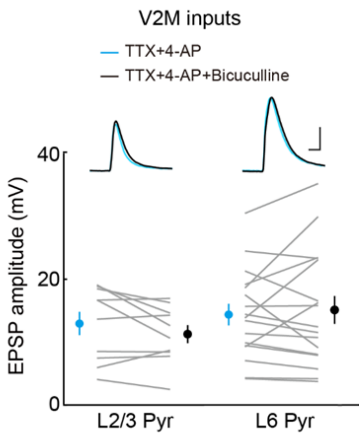

Supplementary Fig. 2. Antagonist of GABA$_A$ receptors has no effect on the EPSP amplitude of V2M inputs on V1 Pyrs. Top, example EPSPs recorded with (black) and without (blue) bicuculline. Bottom, bath application of bicuculline (a GABA$_A$ receptor antagonist, 20 μM), in the presence of TTX and 4-AP (blocking local neuron spikes), caused no significant changes in the EPSP amplitudes of V2M inputs on both L2/3 and L6 Pyrs in V1. L2/3 Pyrs, n = 4 mice, 10 slices, 10 neurons, P = 0.28, Wilcoxon signed-rank test. L6 Pyrs, n = 4 mice, 17 slices, 17 neurons, P = 0.79. Scale bars, 5 mV, 25 ms. Data are presented as the mean ± SEM. Source data are provided as a Source Data file.

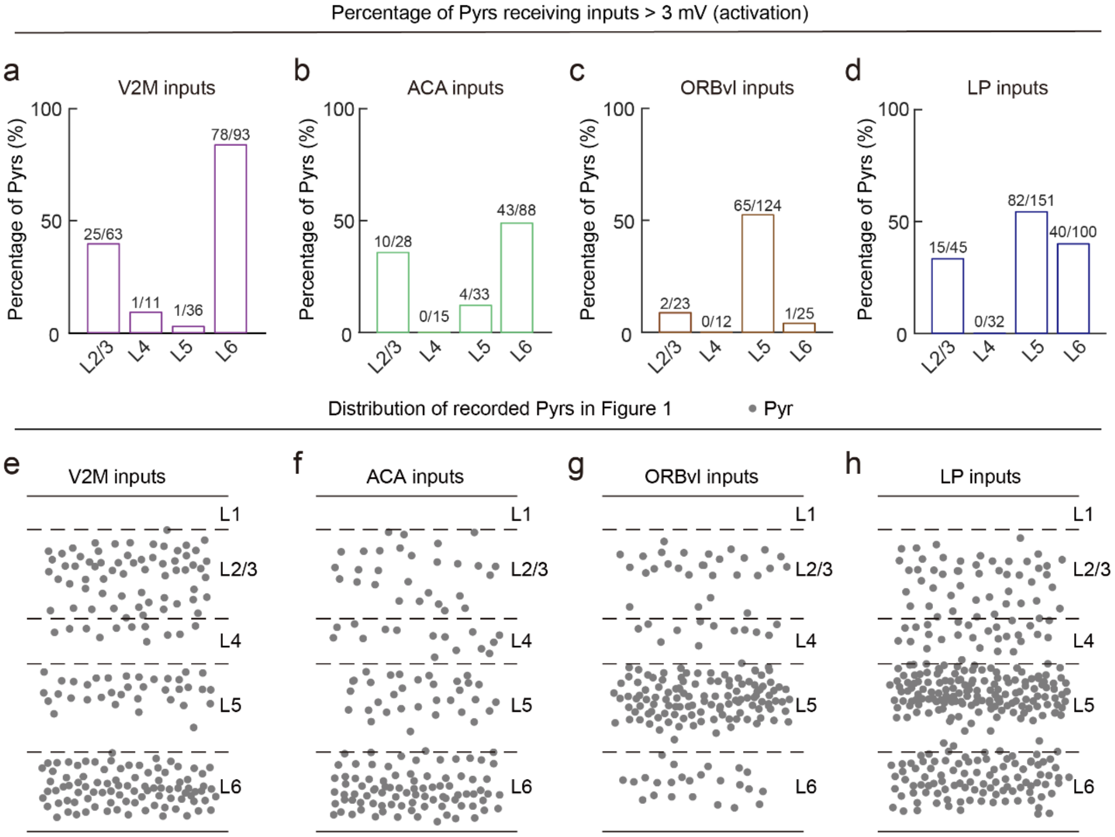

Supplementary Fig. 3. Proportion of V1 Pyrs activated by each CC and TC input across different layers and the distribution of recorded V1 Pyrs. a, Percentage of Pyrs in V1 receiving V2M inputs exceeding 3 mV across different layers. **b**, Similar to **a**, but for ACA inputs. **c**, Similar to **a**, but for ORBvl inputs. **d**, Similar to **a**, but for LP inputs. **e**, Distribution of recorded Pyrs in experiments involving optogenetic activation of V2M inputs. **f-h**, Similar to **e**, but for the experiments involving optogenetic activation of ACA, ORBvl, and LP inputs. Source data are provided as a Source Data file.

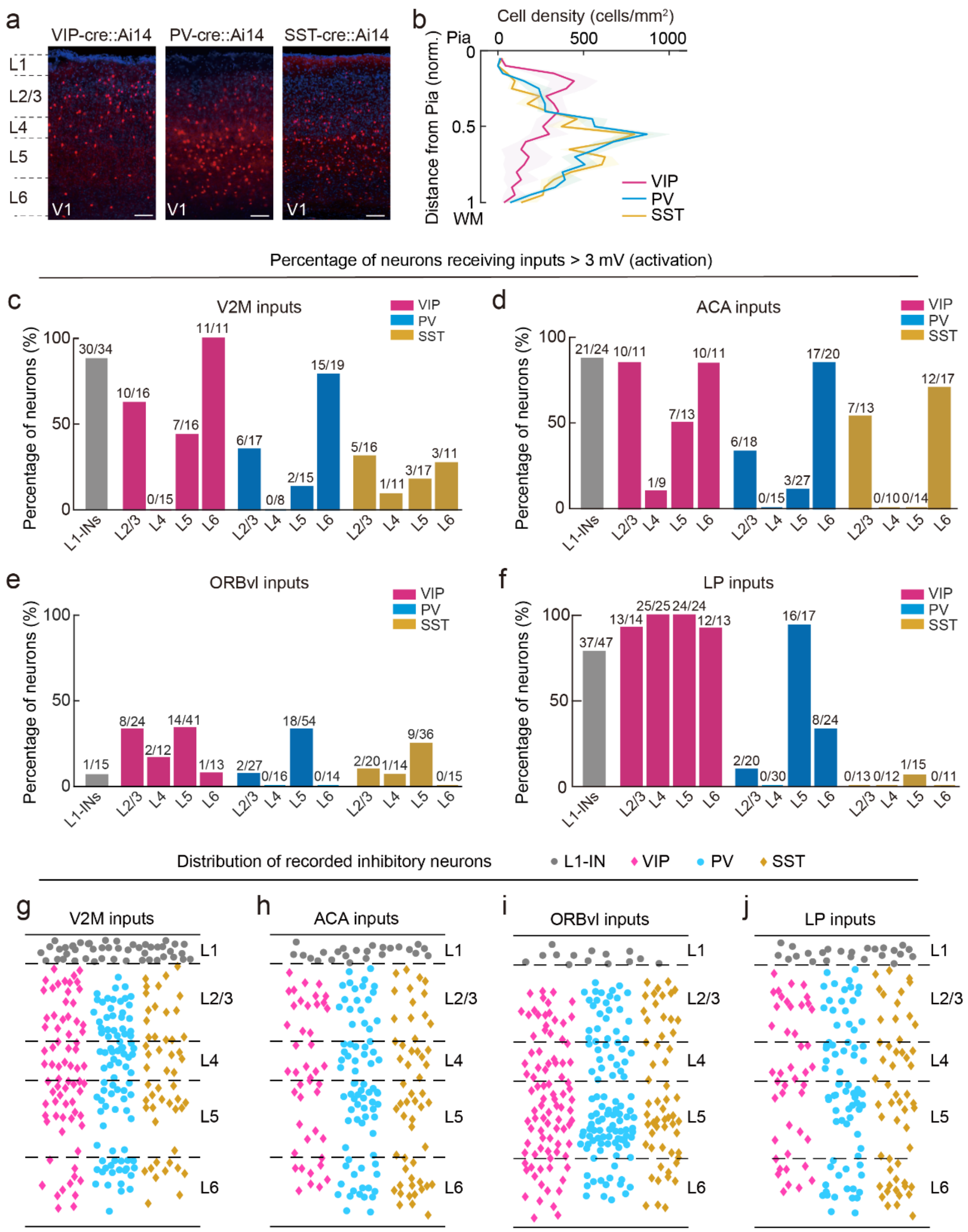

Supplementary Fig. 4. Proportion of V1 inhibitory neurons activated by each CC and TC input across different layers and the distribution of recorded V1 inhibitory neurons. a, Fluorescent images showing the distribution of VIP+, PV+ and SST+ neurons across different layers in V1. Red, VIP+, PV+, and SST+ neurons expressing tdTomato. Blue, DAPI. Scale bar, 100 μm. **b**, The cell density of VIP+ (red), PV+ (blue) and SST+ (yellow) neurons across layers in V1. Colored shading, ±SEM. VIP+ neurons, n = 5 mice; PV+ neurons, n = 6; SST+ neurons,

n = 4. **c**, Percentage of four types of inhibitory neurons (L1-INs, VIP+, PV+, and SST+ neurons) in V1 receiving V2M inputs exceeding 3 mV in each layer. **d**, Similar to **c**, but for ACA inputs. **e**, Similar to **c**, but for ORBvl inputs. **f**, Similar to **c**, but for LP inputs. The number of neurons in each group is indicated by the numbers displayed in the figure. **g**, Distribution of recorded inhibitory neurons in experiments involving optogenetic activation of V2M inputs. **h-j**, Similar to **g**, but for experiments involving optogenetic activation of ACA, ORBvl, and LP inputs. Source data are provided as a Source Data file.

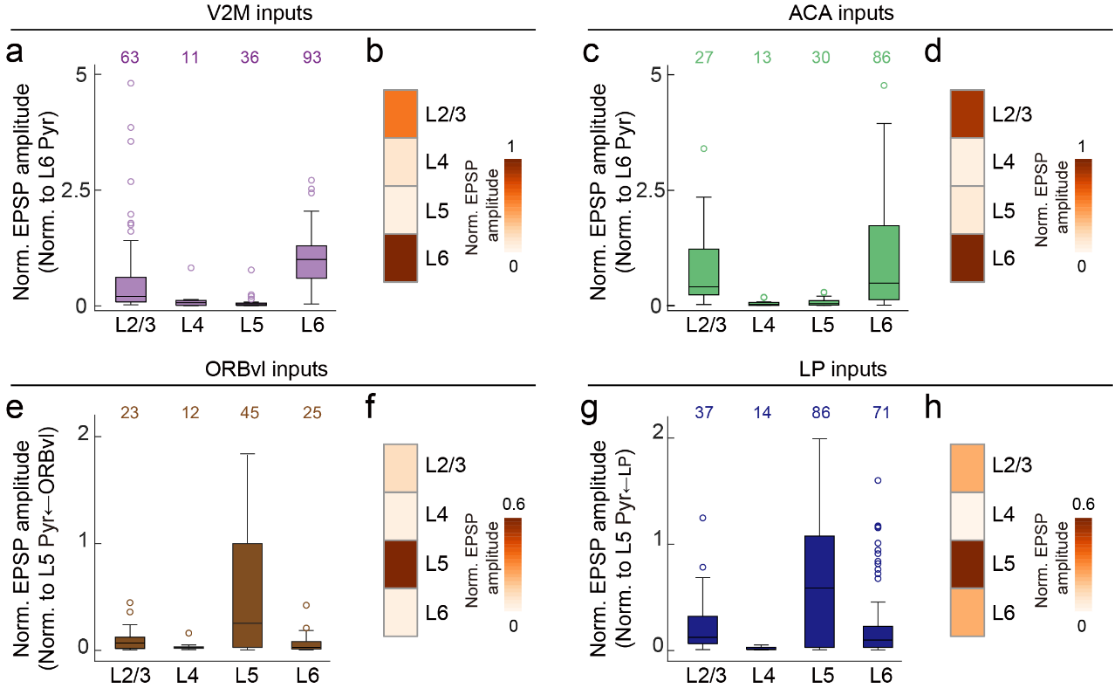

Supplementary Fig. 5. Layer specificity of CC and TC top-down inputs based on normalized EPSP amplitude. a, Distribution of normalized EPSP amplitude from Pyrs in different layers. Edges, 25th and 75th percentiles; central line, median; whiskers, 1.5× the interquartile range of the edges. Circles are outliers, defined as values more than 3 times the interquartile range (IQR) from the median. V2M inputs activated the Pyrs in L2/3 and L6, with the strongest input strength in L6 (significant differences were observed across layers $F(3,199) = 20.7$, $P = 1 \times 10^{-11}$, one-way ANOVA; L6 vs. other layers, $P < 0.002$, Tukey's post hoc test). **b**, Matrix of normalized EPSP amplitude in Pyrs across layers of V1 for V2M inputs. **c-d**, Similar to **a-b**, but for ACA inputs. ACA inputs activated the Pyrs in L2/3 and L6, with stronger input strength in L6 (significant differences were observed across layers, $F(3,152) = 10.2$, $P = 4 \times 10^{-6}$, one-way ANOVA; L6 vs. L4 and L5, $P < 0.004$; L6 vs. L2/3, $P = 0.87$, Tukey's post hoc test). **e-f**, Similar to **a-b**, but for ORBvl inputs. ORBvl inputs only activated the Pyrs in L5 (significant differences were observed across layers, $F(3,101) = 13.4$, $P = 2 \times 10^{-7}$, one-way ANOVA; L5 vs. other layers, $P < 5 \times 10^{-4}$, Tukey's post hoc test). **g-h**, Similar to **a-b**, but for LP inputs. LP inputs activated the Pyrs in L2/3, L5, and L6, with the strongest input strength in L5 (significant differences were observed across layers, $F(3,204) = 15.8$, $P = 3 \times 10^{-9}$, one-way ANOVA; L5 vs. other layers, $P < 2 \times 10^{-5}$, Tukey's post hoc test). The number of neurons in each group is indicated by the numbers displayed in the figure. The EPSP amplitudes of V2M and ACA inputs were

normalized to the averaged L6 Pyr EPSP amplitude recorded the same day. Given the observed heterogeneity in L5 Pyr subtypes responding to ORBvl and LP inputs, we normalized the EPSP amplitudes of ORBvl and LP inputs to the average for $\text{Pyr}_{\leftarrow \text{ORBvl}}$ and for $\text{Pyr}_{\leftarrow \text{LP}}$ neurons. The included data are from Pyrs recorded from at least two layers in the same brain slice, including the layer with the strongest response (L6 for V2M and ACA inputs, and L5 for ORBvl and LP inputs). Source data are provided as a Source Data file.

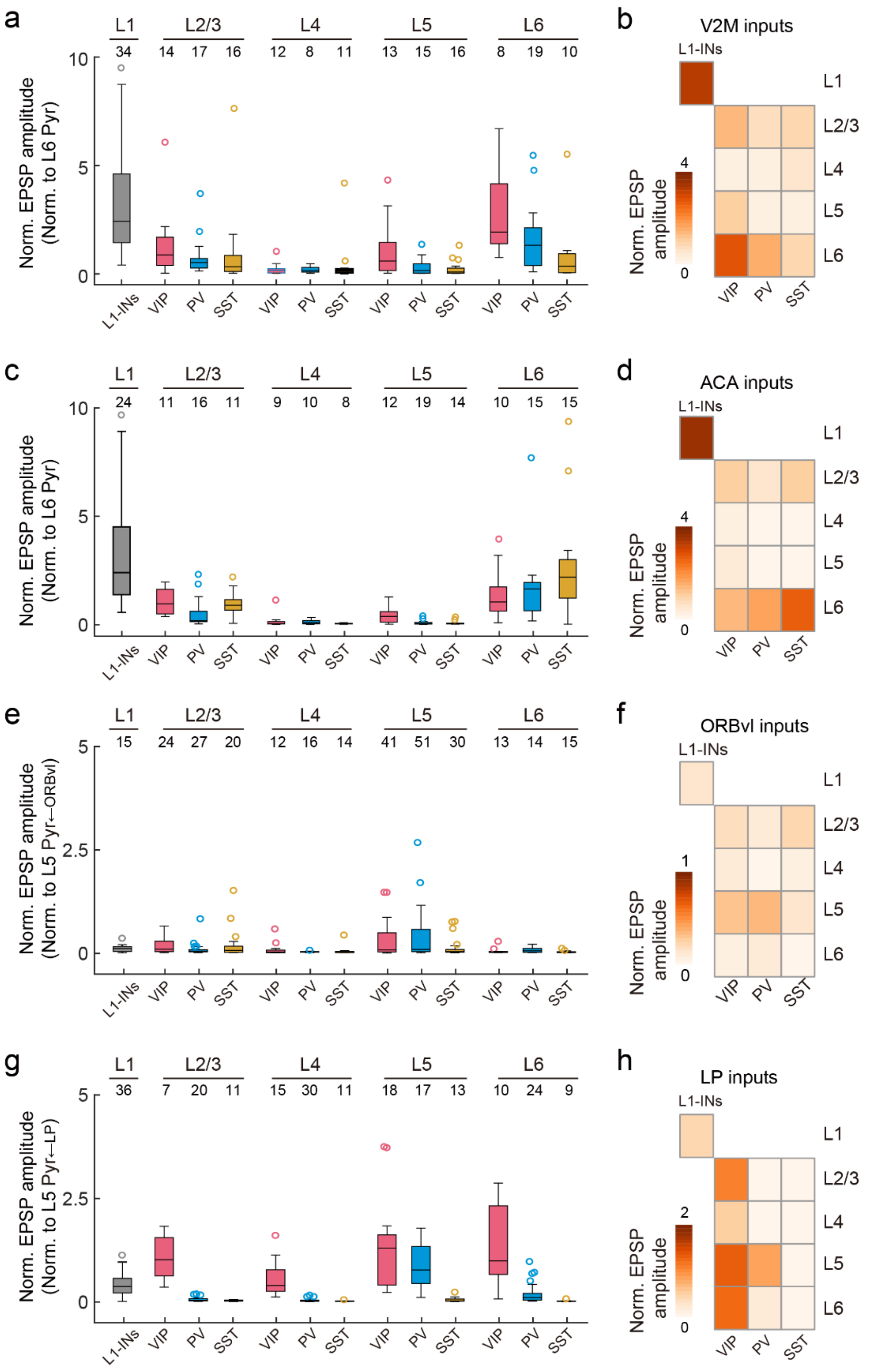

Supplementary Fig. 6. Normalized input strengths of CC and TC top-down inputs in four types of V1 inhibitory neurons across different layers. a, Distribution of normalized EPSP amplitude from inhibitory neurons in different layers. Box plots indicate the median and the respective quartiles. Circles are outliers, defined as values above 3 IQR from the median. **b**,

Matrix of normalized EPSP amplitude in different types of inhibitory neurons across layers of V1 for V2M inputs. **c-d**, Similar to **a-b**, but for ACA inputs. **e-f**, Similar to **a-b**, but for ORBvl inputs. **g-h**, Similar to **a-b**, but for LP inputs. The number of neurons in each group is indicated by the numbers displayed in the figure. The EPSP amplitudes of V2M and ACA inputs were normalized to the averaged L6 Pyr EPSP amplitude recorded the same day. Given the observed heterogeneity in L5 Pyr subtypes responding to ORBvl and LP inputs, we normalized the EPSP amplitudes of ORBvl and LP inputs to the average for $Pyr_{\leftarrow ORBvl}$ and for $Pyr_{\leftarrow LP}$ neurons. The data included are those with Pyrs recorded from the most responsive layer in the same brain slice. Source data are provided as a Source Data file.

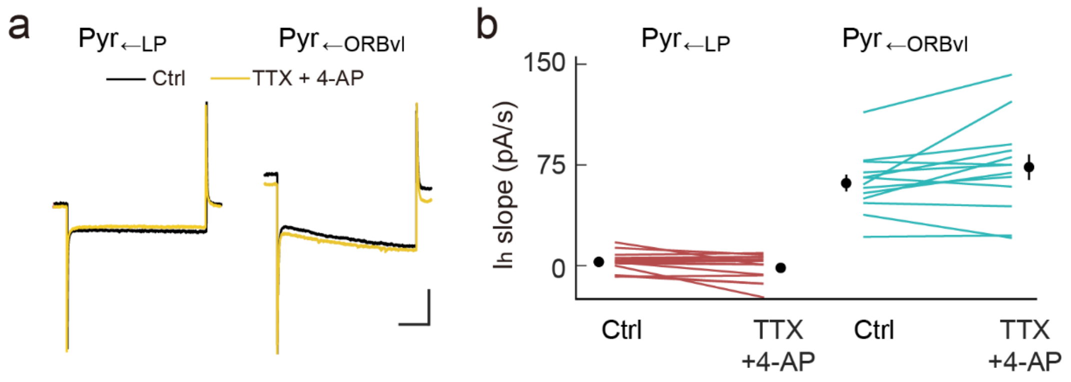

Supplementary Fig. 7. Bath application of antagonists of voltage-gated sodium and potassium channels (TTX and 4-AP) had no effect on I_h slope. a, Left, current change elicited by a 10-mV hyperpolarization in an example $Pyr_{\leftarrow LP}$ neuron with (yellow) or without (black) TTX and 4-AP. Right, similar to left, but for an example $Pyr_{\leftarrow ORBvl}$ neuron. Scale bars, 100 ms, 50 pA. **b**, TTX and 4-AP had no effect on I_h slope of $Pyr_{\leftarrow LP}$ (red) and $Pyr_{\leftarrow ORBvl}$ (green) neurons. $Pyr_{\leftarrow LP}$, n = 13 neurons, 13 slices， 3 mice, P = 0.12; $Pyr_{\leftarrow ORBvl}$, n = 14 neurons, 14 slices, 3 mice, P = 0.07; two-sided Wilcoxon signed-rank test. Data are presented as the mean ± SEM. Source data are provided as a Source Data file.

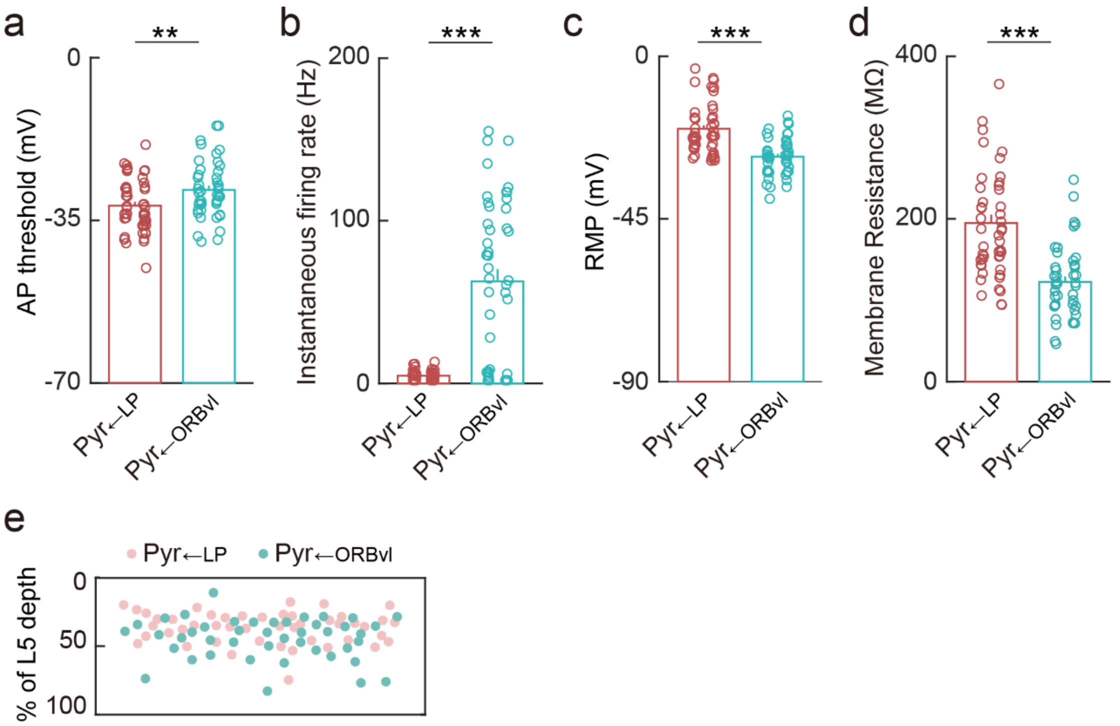

Supplementary Fig. 8. Electrophysiological properties of Pyr$_{\leftarrow \mathrm{LP}}$ and Pyr$_{\leftarrow \mathrm{ORBvl}}$ neurons. a, The AP threshold of Pyr$_{\leftarrow \mathrm{LP}}$ and Pyr$_{\leftarrow \mathrm{ORBvl}}$ neurons. Pyr$_{\leftarrow \mathrm{LP}}$, n = 47 neurons; Pyr$_{\leftarrow \mathrm{ORBvl}}$, n = 44 neurons; P = 0.008, two-sided Wilcoxon rank-sum test. **b**, Similar to **a**, but for the instantaneous firing rate of the first two APs elicited by positive current injection. $P = 2 \times 10^{-7}$. **c**, Similar to **a**, but for the resting membrane potential. $P = 5 \times 10^{-7}$. **d**, Similar to **a**, but for the input resistance. $P = 8 \times 10^{-8}$. Data are presented as the mean ± SEM. **P < 0.01, ***P < 0.001. **e**, Distribution of recorded Pyr$_{\leftarrow \mathrm{LP}}$ and Pyr$_{\leftarrow \mathrm{ORBvl}}$ neurons. Source data are provided as a Source Data file.

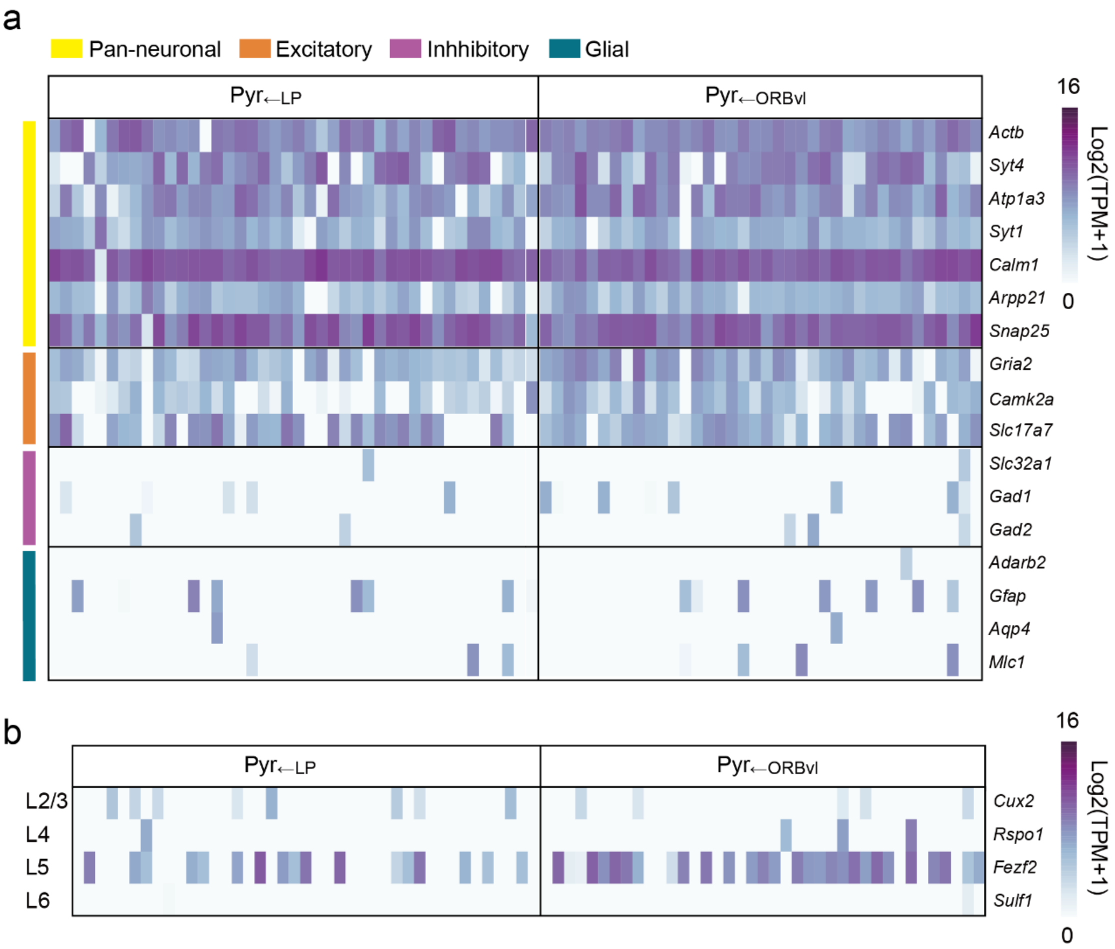

Supplementary Fig. 9. Gene expression profiles of known marker genes in Pyr$_{\leftarrow LP}$ and Pyr$_{\leftarrow ORBvl}$ neurons. a, Heat map illustrating gene expression profiles of pan-neuronal, excitatory, inhibitory, and glial markers in examined Pyr$_{\leftarrow LP}$ and Pyr$_{\leftarrow ORBvl}$ neurons. Pyr$_{\leftarrow LP}$, n = 41 neurons; Pyr$_{\leftarrow ORBvl}$ n = 39 neurons. **b**, Similar to **a**, but for layer-specific genes.

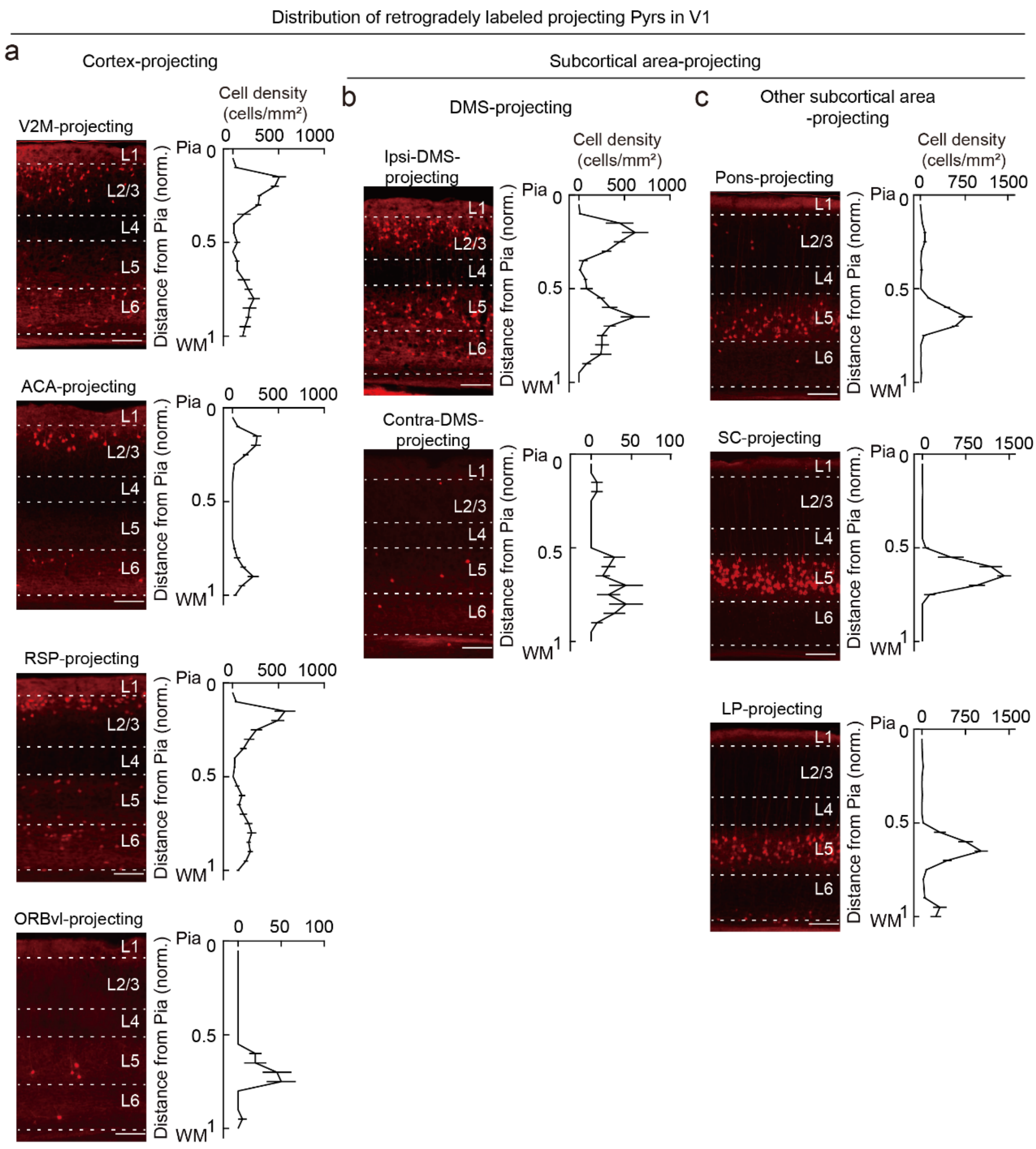

Supplementary Fig. 10. Distribution of retrogradely labeled projecting Pyrs in V1. a, Distribution of retrogradely labeled V1 projecting Pyrs following injection of a Retro-AAV into various cortical areas, including the V2M, ACA, RSP, and ORBvl. Left, Fluorescence images showing the distribution of retrogradely labeled projecting Pyrs (red) in V1. Right, the cell density of labeled projecting Pyrs across different layers in V1. Error bar, ±SEM. V2M, n = 3 mice; ACA, n = 3 mice; RSP, n = 3 mice; ORBvl, n = 3 mice. **b**, Similar to **a**, but for retrogradely labeled V1 projecting Pyrs following Retro-AAV injection into the ipsi- and contra-DMS. Ipsi-DMS, n = 3 mice; contra-DMS, n = 3 mice. **c**, Similar to **a**, but for retrogradely labeled V1 projecting Pyrs following Retro-AAV injection into the Pons, SC, and LP). Pons, n = 3 mice; SC,

n = 3 mice; LP, n = 3 mice. Scale bars, 100 μm. Source data are provided as a Source Data file.

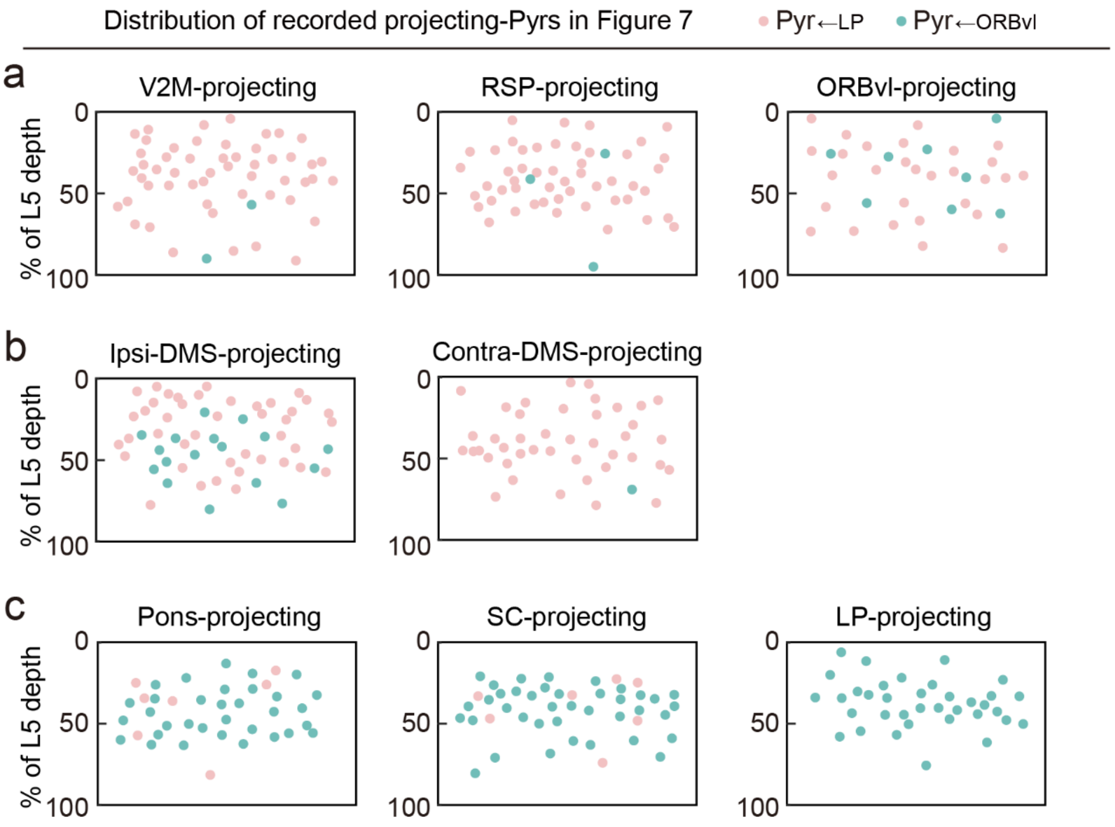

Supplementary Fig. 11. Distribution of recorded projecting-Pyrs in L5 of V1 shown in Figure 7. a, Distribution of recorded cortex-projecting Pyrs. **b**, Similar to **a**, but for DMS-projecting Pyrs. **c**, Similar to **a**, but for other subcortical area-projecting Pyrs. Source data are provided as a Source Data file.

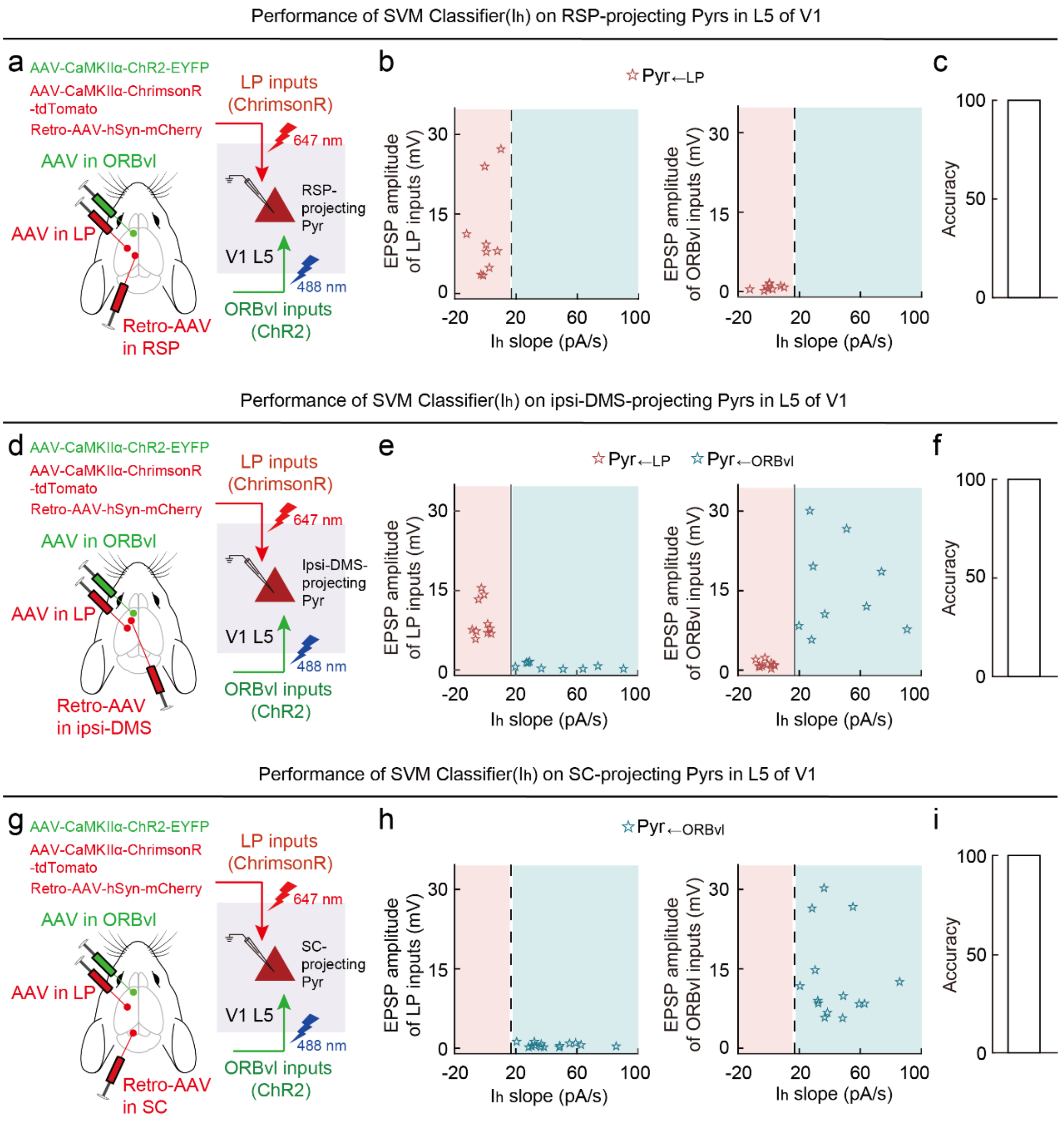

Supplementary Fig. 12. Performance of SVM Classifier(I_h) in distinguishing between $\text{Pyr}_{\leftarrow LP}$ and $\text{Pyr}_{\leftarrow ORBvl}$ neurons in various groups of retrogradely labeled V1 projecting-Pyrs. a, Schematic of the viral strategy (left) and the slice experiment (right) for examination of LP and ORBvl inputs in V1 L5 SC-projecting Pyrs. **b**, Performance of the SVM Classifier(I_h) in distinguishing $\text{Pyr}_{\leftarrow LP}$ from $\text{Pyr}_{\leftarrow ORBvl}$ neurons in the dataset of RSP-projecting Pyrs (the neuron types were initially defined based on their input patterns as determined upon independent optogenetic activation of LP and ORBvl inputs). Dashed line, decision boundary for classifying $\text{Pyr}_{\leftarrow LP}$ and $\text{Pyr}_{\leftarrow ORBvl}$ neurons. Red stars, input-pattern-classified $\text{Pyr}_{\leftarrow LP}$ neurons. n = 3 mice, 3 slices, 9 neurons. **c**, Prediction accuracy. **d-f**, Similar to **a-c**, but for the performance of SVM Classifier(I_h) on ipsi-DMS-projecting Pyrs. Red and green stars, input-pattern-classified $\text{Pyr}_{\leftarrow LP}$

and $Pyr_{\leftarrow ORBvl}$ neurons. n = 3 mice, 3 slices, 19 neurons. **g-i**, Similar to **a-c**, but for the performance of SVM Classifier(I_h) on SC-projecting Pyrs. Green stars, input-pattern-classified $Pyr_{\leftarrow ORBvl}$ neurons. n = 4 mice, 4 slices, 14 neurons. In these three groups of L5 projecting Pyrs, the SVM Classifier(I_h) achieves 100% prediction accuracy in distinguishing $Pyr_{\leftarrow LP}$ from $Pyr_{\leftarrow ORBvl}$ neurons. Source data are provided as a Source Data file. Supplementary Figure 12, Panels a, d, and g adapted from Petrucco, L. (2020). Mouse head schema. Zenodo. https://doi.org/10.5281/zenodo.3925903 under a CC BY license: https://creativecommons.org/licenses/by/4.0/.

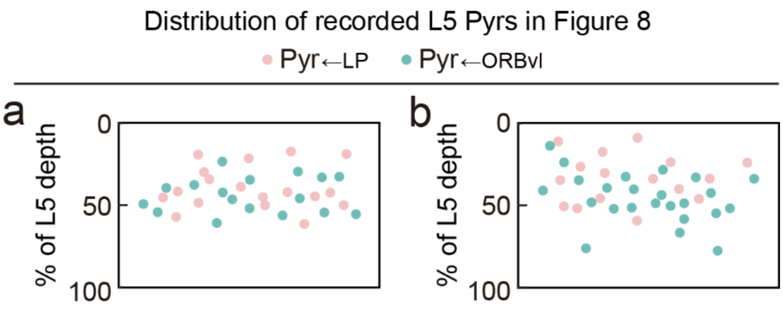

Supplementary Fig. 13. Distribution of recorded Pyr$_{\leftarrow LP}$ and Pyr$_{\leftarrow ORBvl}$ neurons in Figure 8. **a**, Distribution of recorded Pyr$_{\leftarrow LP}$ and Pyr$_{\leftarrow ORBvl}$ neurons in Fig.8b. **b**, Similar to **a**, but for recorded Pyr$_{\leftarrow LP}$ and Pyr$_{\leftarrow ORBvl}$ neurons in Fig.8g. Source data are provided as a Source Data file.

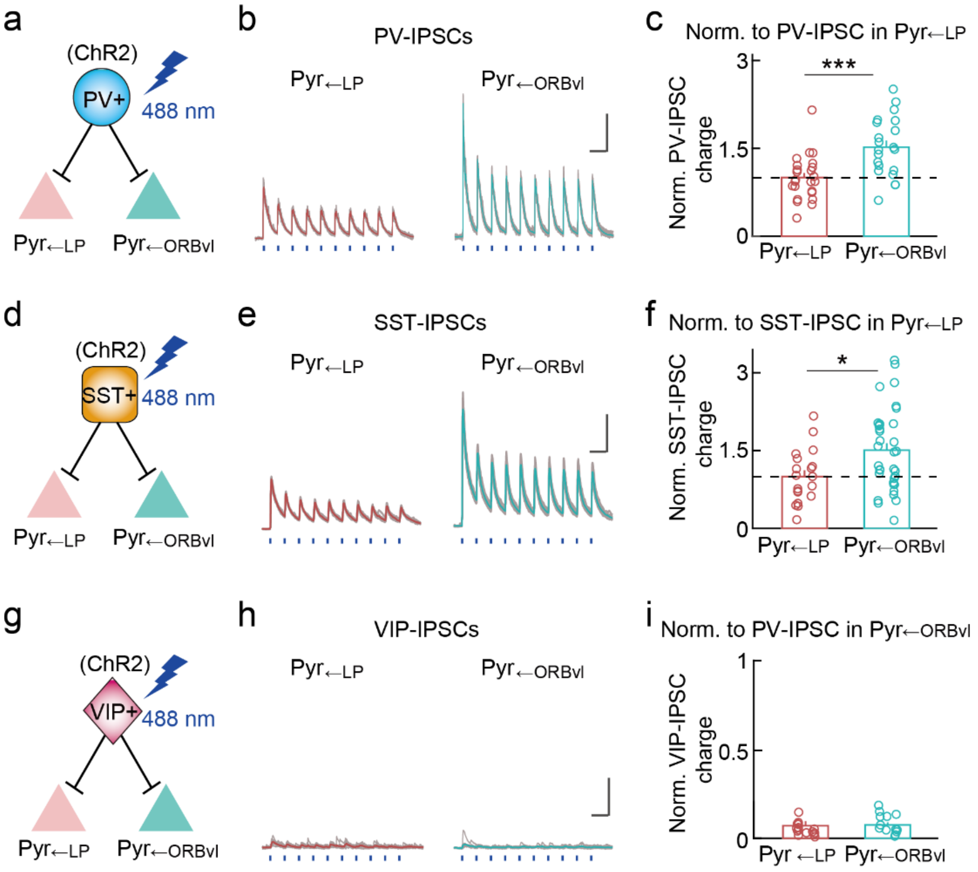

Supplementary Fig. 14. Inhibition on Pyr$_{\leftarrow LP}$ and Pyr$_{\leftarrow ORBvl}$ neurons induced by various cortical inhibitory neuron types. a, Schematic of the slice experiment to measure PV+ neuron-induced IPSCs in Pyr$_{\leftarrow LP}$ and Pyr$_{\leftarrow ORBvl}$ neurons. **b**, Example PV-IPSCs recorded from an example Pyr$_{\leftarrow LP}$ neuron (red) and an example Pyr$_{\leftarrow ORBvl}$ neuron (green). Gray traces, raw traces; colored traces, averaged traces. Blue dots, 5-ms blue light stimulation (474 nm, 10 Hz, 3.5 mW). Scale bars, 100 ms, 500 pA. **c**, Normalized PV-IPSC amplitudes in Pyr$_{\leftarrow ORBvl}$ neurons were significantly larger than in Pyr$_{\leftarrow LP}$ neurons. n = 3 mice, 4 slices. Pyr$_{\leftarrow LP}$, n = 24 neurons, Pyr$_{\leftarrow ORBvl}$, n = 21 neurons, P = 6 × 10^{-4}, two-sided Wilcoxon rank-sum test. **d-f**, Similar to **a-c**, but for SST+ neuron-induced IPSCs. Normalized SST-IPSC amplitudes in Pyr$_{\leftarrow ORBvl}$ neurons were significantly larger than in Pyr$_{\leftarrow LP}$ neurons. n = 5 mice, 7 slices. Pyr$_{\leftarrow LP}$, n = 19 neurons, Pyr$_{\leftarrow ORBvl}$, n = 35 neurons, P = 0.02, two-sided Wilcoxon rank-sum test. **g-i**, Similar to **a-c**, but for VIP+ neuron-induced IPSCs. Activation of VIP+ neurons evoked only very weak IPSCs in both types, each constituting less than 8% of the IPSCs induced by PV+ neuron in Pyr$_{\leftarrow ORBvl}$ neurons. n = 4 mice, 5 slices. Pyr$_{\leftarrow LP}$, n = 16 neurons, Pyr$_{\leftarrow ORBvl}$, n = 13 neurons. Source data are provided as a Source Data file.

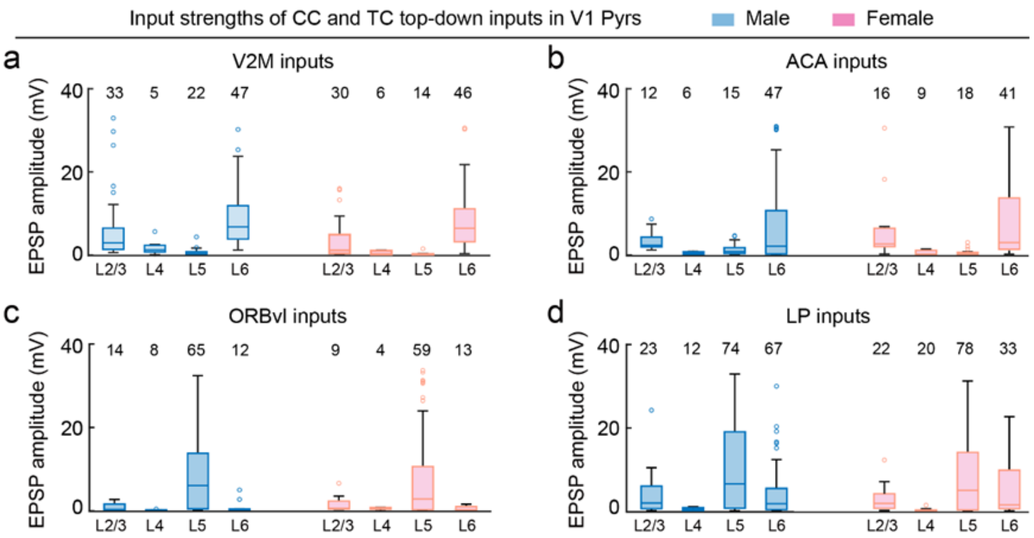

Supplementary Fig. 15. Similar innervation patterns of CC and TC top-down inputs in V1 Pyrs across different layers in both male and female mice. a, EPSP amplitudes evoked by optogenetic activation of V2M inputs in Pyrs across different layers in male (blue) and female (pink) mice. Box plot showing distribution of EPSP amplitude from Pyrs in the indicated layers. Edges, 25th and 75th percentiles; central line, median; whiskers, 1.5× the interquartile range of the edges. Circles are outliers, defined as values more than 3 times the interquartile range (IQR) from the median. No significant sex effect was observed for the input strengths of V2M inputs in V1 Pyrs across different layers ($F_{sex}(1,195) = 1.2$, $P_{\mathrm{sex}} = 0.3$; $F_{sex*layer}(3,195) = 0.7$, $P_{\mathrm{sex*layer}} = 0.6$; two-way ANOVA). **b**, Similar to **a**, but for ACA inputs. No significant sex effect was observed for the input strengths of ACA inputs in V1 Pyrs across different layers ($F_{sex}(1,156) = 0.2$, $P_{\mathrm{sex}} = 0.7$; $F_{sex*layer}(3,156) = 0.2$, $P_{\mathrm{sex*layer}} = 0.9$; two-way ANOVA). **c**, Similar to **a**, but for ORBvl inputs. No significant sex effect was observed for the input strengths of ORBvl inputs in V1 Pyrs across different layers ($F_{sex}(1,176) = 0.02$, $P_{\mathrm{sex}} = 0.9$; $F_{sex*layer}(3,176) = 0.03$, $P_{\mathrm{sex*layer}} = 0.99$; two-way ANOVA). **d**, Similar to **a**, but for LP inputs. No significant sex effect was observed for the input strengths of LP inputs in V1 Pyrs across different layers ($F_{sex}(1,321) = 0.1$, $P_{\mathrm{sex}} = 0.7$; $F_{sex*layer}(3,321) = 0.3$, $P_{\mathrm{sex*layer}} = 0.8$; two-way ANOVA). The number of neurons in each group is indicated by the numbers displayed in the figure. Source data are provided as a Source Data file.

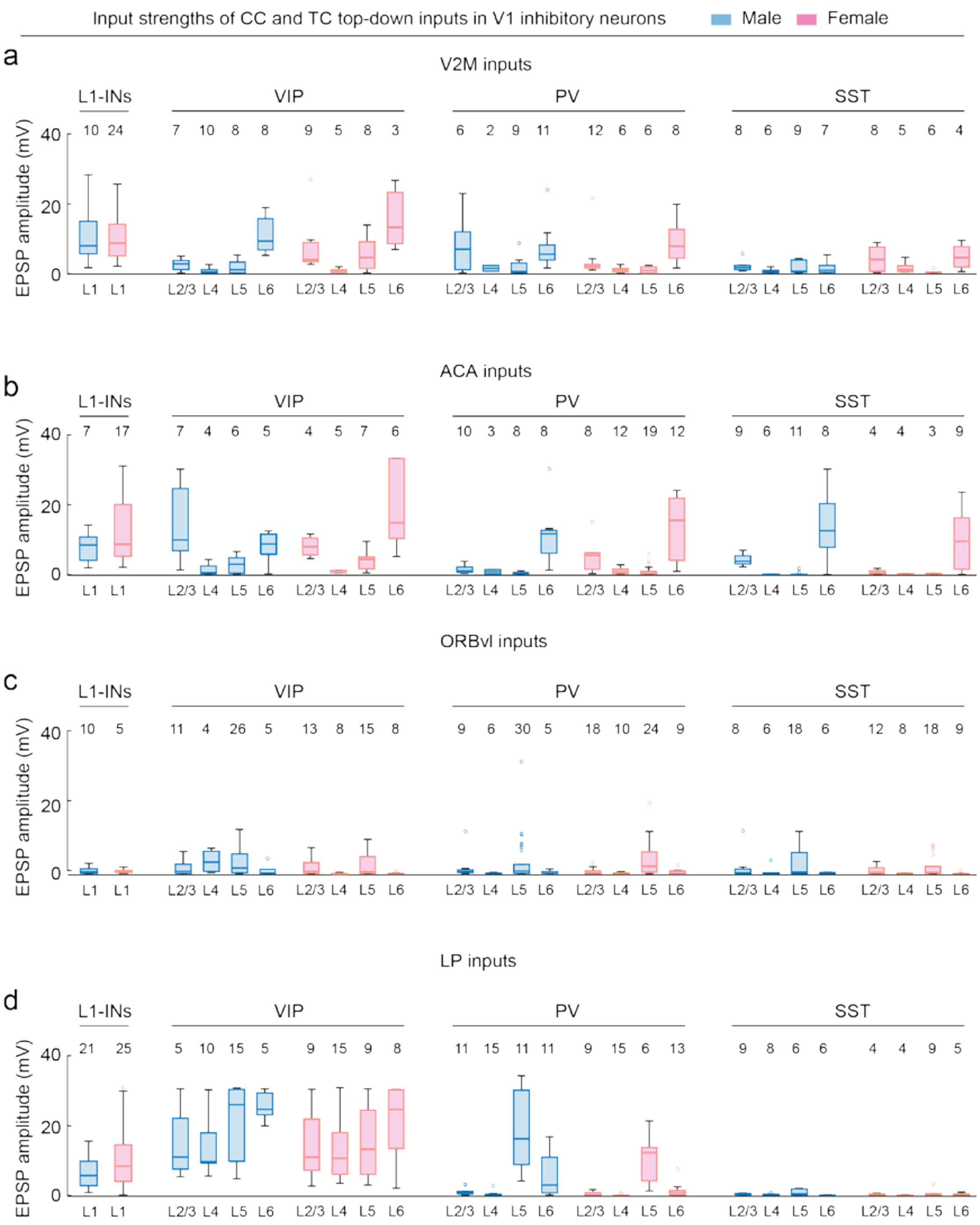

Supplementary Fig. 16. Similar innervation patterns of CC and TC top-down inputs in V1 inhibitory neurons across different layers in both male and female mice. a, EPSP amplitudes evoked by optogenetic activation of V2M inputs in inhibitory neurons across different layers in male (blue) and female (pink) mice. Box plot showing the distribution of EPSP amplitudes from Pyrs in the indicated layers. No significant sex effect was observed for the input strengths of V2M inputs in V1 inhibitory neurons across different layers ($F_{sex}(1,180)$ = 1.5, P_{sex} = 0.2; $F_{sex*layer}(3,180)$ = 0.6, $P_{\mathrm{sex*layer}}$ = 0.6; $F_{sex*cell\text{-}type}(2,180)$ = 2.3, $P_{\mathrm{sex*cell\text{-}type}}$ = 0.1; $F_{sex*layer*cell\text{-}}$

$_{type}(6,180) = 0.7$, $P_{sex*layer*cell\text{-}type} = 0.7$; three-way ANOVA). **b**, Similar to **a**, but for ACA inputs. No significant sex effect was observed for the input strengths of ACA inputs in V1 inhibitory neurons across different layers ($F_{sex}(1,176) = 0.4$, $P_{sex} = 0.6$; $F_{sex*layer}(3,176) = 0.6$, $P_{sex*layer} = 0.6$; $F_{sex*cell\text{-}type}(2,176) = 0.7$, $P_{sex*cell\text{-}type} = 0.5$; $F_{sex*layer*cell\text{-}type}(6,176) = 1.8$, $P_{sex*layer*cell\text{-}type} = 0.1$; three-way ANOVA). **c**, Similar to **a**, but for ORBvl inputs. No significant sex effect was observed for the input strengths of ORBvl inputs in V1 inhibitory neurons across different layers ($F_{sex}(1,275) = 1.1$, $P_{sex} = 0.3$; $F_{sex*layer}(3,275) = 0.4$, $P_{sex*layer} = 0.8$; $F_{sex*cell\text{-}type}(2,275) = 0.4$, $P_{sex*cell\text{-}type} = 0.7$; $F_{sex*layer*cell\text{-}type}(6,275) = 0.5$, $P_{sex*layer*cell\text{-}type} = 0.8$; three-way ANOVA). **d**, Similar to **a**, but for LP inputs. No significant sex effect was observed for the input strengths of LP inputs in V1 inhibitory neurons across different layers ($F_{sex}(1,238) = 1.1$, $P_{sex} = 0.3$; $F_{sex*layer}(3,238) = 1.4$, $P_{sex*layer} = 0.3$; $F_{sex*cell\text{-}type}(2,238) = 0.9$, $P_{sex*cell\text{-}type} = 0.4$; $F_{sex*layer*cell\text{-}type}(6,238) = 0.3$, $P_{sex*layer*cell\text{-}type} = 0.9$; three-way ANOVA). The number of neurons in each group is indicated by the numbers displayed in the figure. Source data are provided as a Source Data file.

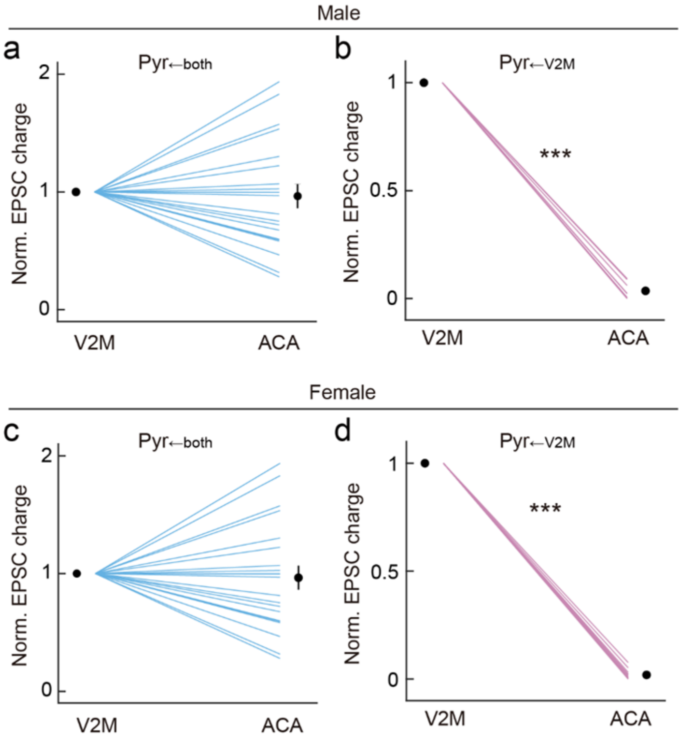

Supplementary Fig. 17. Similar innervation patterns of V2M and ACA inputs in V1 L6 Pyrs in both male and female mice. a-b, Normalized EPSC charge evoked by activation of V2M and ACA inputs in $\text{Pyr}_{\leftarrow\text{both}}$ (a) and $\text{Pyr}_{\leftarrow\text{V2M}}$ (b) neurons in male mice. The circles indicate the means. $\text{Pyr}_{\leftarrow\text{both}}$, n = 21 neurons; $\text{Pyr}_{\leftarrow\text{V2M}}$, n = 11 neurons. **c-d**, Similar to **a-b**, but for $\text{Pyr}_{\leftarrow\text{both}}$ (c) and $\text{Pyr}_{\leftarrow\text{V2M}}$ (d) neurons in female mice. $\text{Pyr}_{\leftarrow\text{both}}$, n = 16 neurons; $\text{Pyr}_{\leftarrow\text{V2M}}$, n = 15 neurons. No significant sex effect was observed for the normalized input strengths of V2M or ACA inputs in V1 L6 Pyrs (Within-Subject Effects Test: $F_{EPSC*sex}(1,59) = 0.04$, $P_{EPSC*\text{sex}} = 0.83$; $F_{EPSC*sex*CellType}(1,59) = 0.002$, $P_{EPSC*\text{sex*CellType}} = 0.96$; Between-Subject Effects Test: $F_{sex}(1,59) = 0.04$, $P_{\text{sex}} = 0.83$; $F_{sex*CellType}(1,59) = 0.002$, $P_{\text{sex*CellType}} = 0.96$; two-way repeated ANOVA). Source data are provided as a Source Data file.

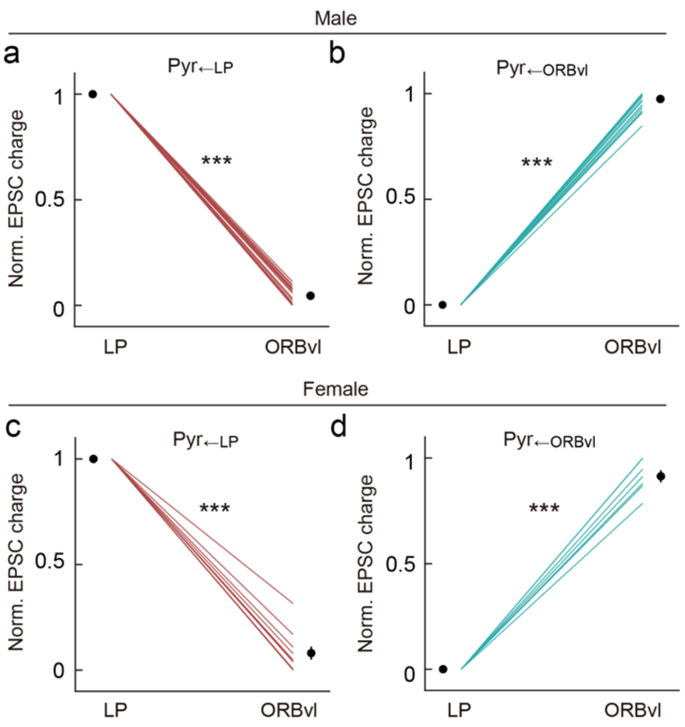

Supplementary Fig. 18. Similar innervation patterns of LP and ORBvl inputs in V1 L5 Pyrs in both male and female mice. a-b, Normalized EPSC charge evoked by activation of LP and ORBvl inputs in $Pyr_{\leftarrow LP}$ (a) and $Pyr_{\leftarrow ORBvl}$ (b) neurons in male mice. The circles indicate the means. $Pyr_{\leftarrow LP}$, n = 18 neurons; $Pyr_{\leftarrow ORBvl}$, n = 28 neurons. **c-d**, Similar to **a-b**, but for $Pyr_{\leftarrow LP}$ (c) and $Pyr_{\leftarrow ORBvl}$ (d) neurons in female mice. $Pyr_{\leftarrow LP}$, n = 10 neurons; $Pyr_{\leftarrow ORBvl}$, n = 7 neurons. No significant sex effect was observed for the input strengths of LP or ORBvl inputs in V1 L5 Pyrs (Within-Subject Effects Test: $F_{EPSC*sex}(1,59) = 0.5$, $P_{EPSC*\mathrm{sex}} = 0.5$; $F_{EPSC*sex*CellType}(1,59) = 0.6$, $P_{EPSC*\mathrm{sex*CellType}} = 0.5$; Between-Subject Effects Test: $F_{sex}(1,59) = 0.3$, $P_{\mathrm{sex}} = 0.6$; $F_{sex*CellType}(1,59) = 0.4$, $P_{\mathrm{sex*CellType}} = 0.5$; two-way repeated ANOVA). Source data are provided as a Source Data file.
