# Supplementary material for: Organization of corticocortical and thalamocortical top-down inputs in the primary visual cortex
Source: Nat Commun. 2024 May 27;15:4495. doi: 10.1038/s41467-024-48924-8 (PMC11130321; doi:10.1038/s41467-024-48924-8)
Supplement: Supplementary file 3 — Description of Additional Supplementary Files [file 41467_2024_48924_MOESM3_ESM.pdf]

Description of Additional Supplementary Files

File name: Supplementary Data 1
Description: The input strengths of CC and TC top-down inputs in V1 Pyrs across different layers.

File name: Supplementary Data 2
Description: Comparison of CC and TC top-down input strengths in V1 Pyrs across different layers. One-way ANOVA was used to compare the input strengths of top-down inputs across layers, followed by a two-sided Tukey's Honestly Significant Difference (HSD) test for pairwise comparisons.

File name: Supplementary Data 3
Description: The input strengths of CC and TC top-down inputs in V1 inhibitory neurons across different layers.

File name: Supplementary Data 4
Description: Comparison of CC and TC top-down input strengths in V1 interneurons across different layers. One-way ANOVA was used to compare the input strengths of top-down inputs across layers, followed by a two-sided Tukey's Honestly Significant Difference (HSD) test for pairwise comparisons.

File name: Supplementary Data 5
Description: Comparison of CC and TC top-down input strengths among different types of interneurons within the same layer of V1. One-way ANOVA was used to compare the input strengths of top-down inputs among different types of interneurons, followed by a two-sided Tukey's Honestly Significant Difference (HSD) test for pairwise comparisons.

File name: Supplementary Data 6
Description: The normalized input strengths of CC and TC top-down inputs in V1 Pyrs across different layers.

File name: Supplementary Data 7
Description: The normalized input strengths of CC and TC top-down inputs in V1 inhibitory neurons across different layers.

File name: Supplementary Data 8
Description: Cell-by-gene count matrix from Patch-seq data collection.

File name: Supplementary Data 9
Description: Cell-by-FPKM value matrix from Patch-seq data collection.

File name: Supplementary Data 10
Description: Metadata for cells included in Patch-seq data collection.

File name: Supplementary Data 11
Description: Differentially expressed genes (DEGs) between Pyr←LP and Pyr←ORBvl neurons. Statistical analysis was performed using the DESeq2 package (version 1.36.0) in R software. Two-sided Wald test was employed to assess the statistical significance of differential expression for each gene. Q-values are P-values adjusted for multiple comparisons using the Benjamini-Hochberg procedure, which controls the false discovery rate (FDR).

File name: Supplementary Data 12
Description: Detailed information on chemicals, peptides, and recombinant proteins used in the current study.

File name: Supplementary Data 13
Description: Number of mice recorded with different numbers of layers in Fig.1.
