# Supplementary material for: Organization of corticocortical and thalamocortical top-down inputs in the primary visual cortex
Source: Nat Commun. 2024 May 27;15:4495. doi: 10.1038/s41467-024-48924-8 (PMC11130321; doi:10.1038/s41467-024-48924-8)
Supplement: Supplementary file 17 — Reporting Summary [file 41467_2024_48924_MOESM17_ESM.pdf]

nature portfolio

Corresponding author(s): Siyu Zhang, Guofen Ma

Last updated by author(s): Apr 16, 2024

Reporting Summary

Nature Portfolio wishes to improve the reproducibility of the work that we publish. This form provides structure for consistency and transparency in reporting. For further information on Nature Portfolio policies, see our Editorial Policies and the Editorial Policy Checklist.

Statistics

For all statistical analyses, confirm that the following items are present in the figure legend, table legend, main text, or Methods section.

n/a	Confirmed	
☐	☒	The exact sample size (n) for each experimental group/condition, given as a discrete number and unit of measurement
☐	☒	A statement on whether measurements were taken from distinct samples or whether the same sample was measured repeatedly
☐	☒	The statistical test(s) used AND whether they are one- or two-sided<br>*Only common tests should be described solely by name; describe more complex techniques in the Methods section.*
☒	☐	A description of all covariates tested
☐	☒	A description of any assumptions or corrections, such as tests of normality and adjustment for multiple comparisons
☐	☒	A full description of the statistical parameters including central tendency (e.g. means) or other basic estimates (e.g. regression coefficient) AND variation (e.g. standard deviation) or associated estimates of uncertainty (e.g. confidence intervals)
☐	☒	For null hypothesis testing, the test statistic (e.g. F, t, r) with confidence intervals, effect sizes, degrees of freedom and P value noted<br>*Give P values as exact values whenever suitable.*
☒	☐	For Bayesian analysis, information on the choice of priors and Markov chain Monte Carlo settings
☒	☐	For hierarchical and complex designs, identification of the appropriate level for tests and full reporting of outcomes
☒	☐	Estimates of effect sizes (e.g. Cohen's d, Pearson's r), indicating how they were calculated

Our web collection on statistics for biologists contains articles on many of the points above.

Software and code

Policy information about availability of computer code

Data collection	The softwares and codes used for anatomic data collection in this study were similar to previous studies (Zhang et al. 2016. Nat. Neurosci.; Ma et al. 2021. Sci. Adv.) and described in Supplementary Materials and Methods. Axon pCLAMP 11 was used for physiological data collection.
Data analysis	Data were analyzed and visualized with MatLab (2014b) and R (4.2.1). ANOVA were conducted with SPSS (IBM SPSS Statistics 25). Adobe Illustrator CS6 were used in the production of data figures.

For manuscripts utilizing custom algorithms or software that are central to the research but not yet described in published literature, software must be made available to editors and reviewers. We strongly encourage code deposition in a community repository (e.g. GitHub). See the Nature Portfolio guidelines for submitting code & software for further information.

Data

Policy information about availability of data

All manuscripts must include a data availability statement. This statement should provide the following information, where applicable:

- Accession codes, unique identifiers, or web links for publicly available datasets
- A description of any restrictions on data availability
- For clinical datasets or third party data, please ensure that the statement adheres to our policy

The data supporting the findings of this study are included in the figures and supporting files. The sequencing data generated in this study have been deposited in

the Gene Expression Omnibus repository under the accession code GSE246589 [https://www.ncbi.nlm.nih.gov/geo/query/acc.cgi?acc=GSE246589]. Source data are provided with this paper.

Research involving human participants, their data, or biological material

Policy information about studies with human participants or human data. See also policy information about sex, gender (identity/presentation), and sexual orientation and race, ethnicity and racism.

Reporting on sex and gender	N/A
Reporting on race, ethnicity, or other socially relevant groupings	N/A
Population characteristics	N/A
Recruitment	N/A
Ethics oversight	N/A

Note that full information on the approval of the study protocol must also be provided in the manuscript.

Field-specific reporting

Please select the one below that is the best fit for your research. If you are not sure, read the appropriate sections before making your selection.

☒ Life sciences ☐ Behavioural & social sciences ☐ Ecological, evolutionary & environmental sciences

For a reference copy of the document with all sections, see nature.com/documents/nr-reporting-summary-flat.pdf

Life sciences study design

All studies must disclose on these points even when the disclosure is negative.

Sample size	No statistical methods were used to predetermine sample size, but the sample size was similar to previous studies (Ma et al.,2021, Sci. Adv; Rindner et al., 2022, Neuron; Ibrahim et al., 2021, Neuron).
Data exclusions	For Patch-seq data analysis, neurons with a low number of detectable genes or a high level of inhibitory neuron markers were excluded. For physiological data analysis, data exclusions were based on the quality of recordings. The recordings were excluded when they terminated too shortly or the series resistance became too high.
Replication	The exact number of repetitions (individual data points from separate cells and/or animal) is indicated in figures and legends. Reproducibility was ensured by sampling from multiple biological replicates, i.e., from three or more mice in each group. No results that were not observed in multiple animals were included.
Randomization	Animals were not randomized due to the necessity of a genetic construct.
Blinding	Investigators were not blind to subject groups because knowledge of experimental conditions was required during data collection and evaluation.

Reporting for specific materials, systems and methods

We require information from authors about some types of materials, experimental systems and methods used in many studies. Here, indicate whether each material, system or method listed is relevant to your study. If you are not sure if a list item applies to your research, read the appropriate section before selecting a response.

Materials & experimental systems

n/a	Involved in the study	
☐	☒	Antibodies
☒	☐	Eukaryotic cell lines
☒	☐	Palaeontology and archaeology
☐	☒	Animals and other organisms
☒	☐	Clinical data
☒	☐	Dual use research of concern
☒	☐	Plants

Methods

n/a	Involved in the study	
☒	☐	ChIP-seq
☒	☐	Flow cytometry
☒	☐	MRI-based neuroimaging

Antibodies

Antibodies used	Morphology construction experiment: Streptavidin, DyLight™ 549 ,Vector Laboratories (cat# SA-5549-1) 1:1000
Validation	Antibodies were validated by the manufacturer-information obtained from the manufacturer's website. For Streptavidin, DyLight™ 549 (https://vectorlabs.com/products/dylight-549-streptavidin/).

Animals and other research organisms

Policy information about studies involving animals; ARRIVE guidelines recommended for reporting animal research, and Sex and Gender in Research

Laboratory animals	We used both wildtype mice (C57) and transgenic mice. The transgenic mouse lines included PV-Cre (Jackson lab stock #017320), SST-Cre (#013044), VIP-Cre (#010908), CaMKIIα-Cre (#005359), loxP-flanked-tdTomato (#007914) mice. The mice used were aged between 2 and 3 months. Mice were housed at 20-24°C with 45 to 65% humidity, adhering to standard laboratory conditions for optimal welfare.
Wild animals	This study did not involve wild animals.
Reporting on sex	Male and female mice were used.
Field-collected samples	This study did not involve samples collected from the field.
Ethics oversight	Animal care and the experimental protocols were approved by the Animal Committee of Shanghai Jiao Tong University School of Medicine and the Animal Committee of the Institute of Neuroscience, Chinese Academy of Sciences.

Note that full information on the approval of the study protocol must also be provided in the manuscript.
